# Thermal manipulation modifies embryonic growth, hepatic free amino acid concentrations, and hatching performance in layer-type chicks

**DOI:** 10.3389/fvets.2022.1049910

**Published:** 2022-11-18

**Authors:** Guofeng Han, Sheng Li, Yansen Li, Phuong V. Tran, Mitsuhiro Furuse, Takashi Bungo, Vishwajit S. Chowdhury, Zongchun Bai, Chunmei Li

**Affiliations:** ^1^Institute of Facilities and Equipment in Agriculture, Jiangsu Academy of Agricultural Sciences, Nanjing, China; ^2^Research Center for Livestock Environmental Control and Smart Production, College of Animal Science and Technology, Nanjing Agricultural University, Nanjing, China; ^3^Department of Animal Nutrition and Feed, National Institute of Animal Sciences, Hanoi, Vietnam; ^4^Laboratory of Regulation in Metabolism and Behavior, Graduate School of Bioresource and Bioenvironmental Sciences, Faculty of Agriculture, Kyushu University, Fukuoka, Japan; ^5^Faculty of Veterinary Medicine, Okayama University of Science, Imabari, Japan; ^6^Laboratory of Stress Physiology and Metabolism, Faculty of Arts and Science, Graduate School of Bioresource and Bioenvironmental Sciences, Kyushu University, Fukuoka, Japan

**Keywords:** amino acid, thermal manipulation, embryo, layer-type, hatching

## Abstract

Thermal manipulation (TM) of incubation temperature has been demonstrated to alter metabolism and post-hatch thermotolerance in broiler strains (meat-type chickens). Fewer reports were focused on layer-type chickens and there was no report on amino acid metabolism during TM in layer-type embryos. In this study, we investigated the effects of TM on embryonic development, hepatic amino acid metabolism, and hatching performance in layer-type chickens. Fertilized eggs were incubated under control thermoneutral temperature (CT, 37.6°C) and TM with high temperature (TMH, 39°C, 8 h/day) or low temperature (TML, 20°C, 1 h/day) from embryonic day (ED) 8 to ED 15. The embryonic weight and relative embryonic weight (yolk-free embryonic weight to the initial egg weight) significantly declined in the TML group at ED 13 (*P* < 0.01) and ED 16 (*P* < 0.0001), and were significantly increased (*P* < 0.001) in the TMH group at ED 16, in comparison with the embryos in the CT group. The concentrations of all hepatic free amino acids were significantly increased (*P* < 0.01) with embryonic development. Interestingly, TMH and TML caused similar effects on hepatic amino acid metabolism, in which most of the essential and non-essential amino acids were significantly declined (*P* < 0.05) under TM treatments at ED 13 but not affected at ED 16. Until hatching, TML, but not TMH, caused a significant (*P* < 0.05) delay (31–38 min/day from ED 8) in incubation duration. The hatchability in the TML group was lower than the other two groups, which indicated that 20°C as cold stimulation was not suitable for layer embryos. The body weight, yolk weight, yolk-free body mass, and chick quality were not affected by TM treatments. However, the relative weight of the liver, but not the heart, was significantly reduced (*P* < 0.05) at hatching by TML treatment. In conclusion, TML, but not TMH, caused to delay in embryogenesis and affected the internal organ of chicks at hatch. Similar changes in amino acid metabolism under TMH and TML indicated that thermal stress induced by both high and low extreme ambient temperatures influences embryonic amino acid metabolism in a similar fashion in layer-type embryos.

## Introduction

Incubation temperature is considered the most critical environmental factor for embryonic development and hatching efficiency (1), as the thermogenesis of embryos is limited and embryos are kept warm by incubators or hens before hatching (2). Over-heated incubation was reported to cause red hocks, unhealed navels, and lower yolk-free chick weight (3). An eggshell temperature of 40°C during the last 5 days of incubation caused hatchability to decline by 10–20% for broiler strains and meat-type chickens (4). Thermal manipulation (TM) means changing the standard incubation temperature during embryogenesis and results in the modification of the performance and fitness of chickens (3). Thermal manipulation (TM) of apposite high incubation temperature, such as 38.5–39.5°C for 6–8 h per day during embryonic days (EDs) 10–18, has been well studied and demonstrated to improve thermotolerance in post-hatch chicks and chickens of broiler strains (1, 5–7), as heat stress is a critical challenge for commercial broiler production in hot summer (8). Higher eggshell temperature (38.5°C) during incubation could improve bone morphology and ash contents of bones in layer strains (9). Improvement of cold resistance is a benefit to reducing ascites syndrome in broilers under cold conditions. A short-term cold exposure (15°C for 30 or 60 min) during incubation was considered to improve the adaptation of thermoregulatory and cardiovascular systems to cold conditions in post-hatch broilers (10). Few studies reported that TM of low temperature or cold exposure during embryogenesis supported birds to cope with low ambient temperature and caused positive effects on growth in post-hatch broilers (11, 12). Cyclic low incubation temperature (36.5°C) modified thyroid activity with an increased plasma thyroxine level under cold stress in post-hatch hens (13). However, few studies focused on the effects of TM on layer-type embryonic development.

Amino acids serve as building blocks of protein, and the free amino acid pool enlarges with embryonic growth during incubation (14). Amino acid administration during incubation was reported to increase body weight in post-hatched chicks and chickens (15). Stress-related research suggested that amino acids also play important roles in stress response and minimizing stress levels in poultry (16). Short- or long-term exposure to heat stress was reported to modify several free amino acids in blood and tissues in layer- and meat-type chicks or chickens (17–20). In our previous study, TM treatment during incubation caused to reduce of several free amino acids, including leucine (Leu) in the brain and the liver of broiler embryos (21), and *in ovo* administration of L-Leu was demonstrated to improve thermotolerance in broiler chicks and chickens (20, 22). However, the studies of amino acid profiles in layer-type embryos were less focused.

The initial body weight corresponded to the growth rate and later body weight in broiler chicks during the post-hatch first week (23). Thermal manipulation (TM) of high incubation temperature was reported to influence the incubation period and organ development in broiler strains (24, 25). Several hatching parameters of day-old chicks were considered predictors of growth potential in broilers (26). The average commercial life span of a layer hen is 72 weeks, which is longer than that of commercial broilers. However, the potential benefit of TM treatments in layer-type chicks was less focused. In the present study, the aims were to investigate the effects of cyclic heat or cold exposure to embryos on embryo growth, changes in hepatic amino acid concentrations, and chick quality in commercial layer strain. It was hypothesized that amino acid profiles as well as the growth of embryos will be modified by TM treatments.

## Materials and methods

### Incubation, thermal manipulation, and experimental design

**Figure 1:**
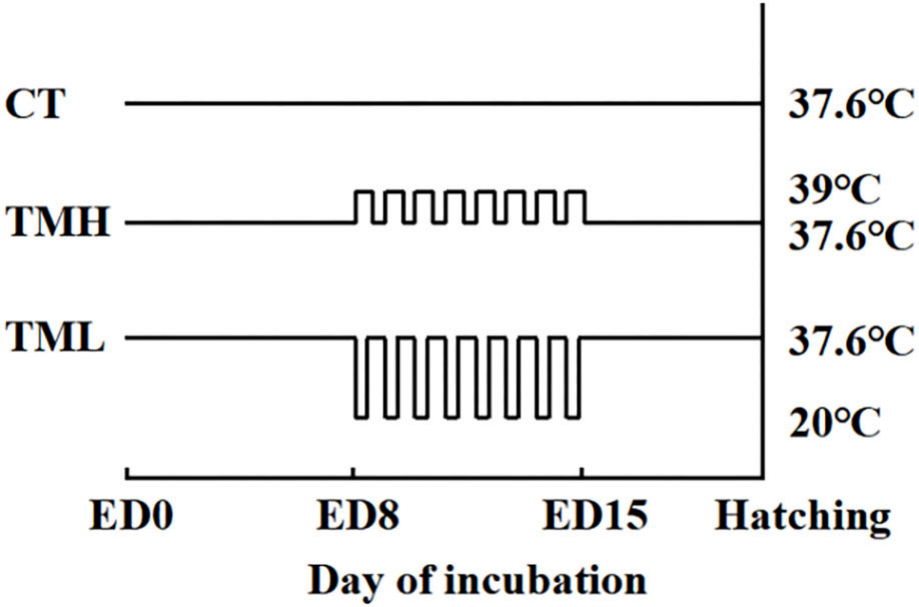
Incubation treatments. CT, control incubation temperature until hatching at 37.6°C; TMH, thermal manipulation with high temperature (39°C, 8 h/day) from embryonic day (ED) 8 to ED 15; TML, thermal manipulation with low temperature (20°C, 1 h/day) from ED 8 to 15. All eggs were transferred to the hatcher at ED 18.

The first experiment (Experiment 1) was conducted to investigate the effects of TM on embryo development and hepatic amino acid changes during embryogenesis. In Experiment 1, 150 fertilized layer eggs (Hy-Line Variety Brown strain; 41 weeks old of parent stock) were purchased from a local hatchery in Jiangsu, China. The eggs were weighted and delivered into three groups (*n* = 50/group): control thermoneutral temperature (CT) group, TM with high temperature (TMH) group, and TM with low temperature (TML) group. The average egg weights (means ± SEM) were 60.72 ± 0.60 g, 60.90 ± 0.67 g, and 60.57 ± 0.65 g for CT, TMH, and TML, respectively. All eggs were placed into an incubator (Hongde 2112 type incubator, Hongde Co., Shandong, China). According to our previous studies, the incubation temperature was 37.6°C with 60–70% relative humidity and auto-turning every 1.5 h (19, 20, 27). On ED 7, all eggs were candled and unfertilized eggs were discarded properly. The number of unfertilized eggs was 9, 4, and 3 for CT, TMH, and TML, respectively. The hypothalamic-pituitary-thyroid (HPT) and hypothalamic-pituitary-adrenal (HPA) axes are responsible for the regulation of metabolism and thermoregulation, as well as response to stress. The HPT and HPA axes mainly develop and mature between ED 7 and 16 during embryogenesis in chicks (7, 28). The incubation temperature between 38.5 and 39.5°C was well tested to afford thermotolerance (1) and a short-term cold exposure at 15°C for 30 min or 60 min was reported to cause no effects on hatchability and body weight at hatching, as well as positive effects on growth in broiler chickens (10). Thus, eggs from the TMH group were exposed to 39°C (29) for 8 h every day and eggs from the TML group were exposed to 20°C for 1 h every day in other two small incubators (Hongde 440 type incubator, Hongde Co., Shandong, China) from ED 8 to 15. A cyclical treatment of 39 or 20°C caused no significant effects on the hatching rate in birds (10, 29). The relative humidity was set around 65% during TM treatments. After TM exposure, eggs from the TMH or TML group were returned to the previous incubator with the CT group (Hongde 2112 type incubator, Hongde Co., Shandong, China). The schedule of TM treatments was shown in Figure 1. To investigate the changes in embryo growth and amino acid concentrations during and after TM treatments, developing embryos (*n* = 15/group) were randomly selected from each group for sampling on ED 13 and 16. The sampling of the embryo was performed by the same person without knowing the grouping information. The yolk free body mass (YFBM) was determined after removing the yolk from the embryo. The relative embryo weight (%) was calculated as the ratio between YFBM (g) and the initial egg weight (g). The percent moisture loss from the egg during the incubation was calculated as follows: % moisture loss = [initial egg weight (g) – sampling egg weight (g)]/the initial egg weight (g) multiplied by 100. After the measurement of embryo weight, the liver was collected, snap frozen using liquid nitrogen, and then stored with plasma samples at −80°C until further analysis. After sampling, the remaining eggs were discarded properly.

The second experiment (Experiment 2) was conducted to investigate the effects of TM on hatching performance in layer chicks. In Experiment 2, 75 fertilized layer eggs (Hy-Line Variety Brown strain; 58 weeks old of parent stock) were purchased from a local hatchery as same as in Experiment 1, and 6 eggs were broken before incubation. On ED 7, 12 unfertilized eggs were detected by candling. The remaining eggs were divided into three groups: CT, TMH, and TML (*n* = 19/group) based on the initial egg weight. The average egg weights (means ± SEM) were 60.04 ± 0.70 g, 60.06 ± 0.83 g, and 60.07 ± 0.76 g for CT, TMH, and TML, respectively. The incubation and TM treatments were the same as described in Experiment 1. At the end of ED 18 (incubation time 432 h), all eggs (*n* = 19/group) were shifted to the hatching trays and the hatching process was recorded by a video system (HIKVISION, Hangzhou, China). For each egg, the incubation duration was defined as the time between setting and hatching (30). After 510 h of incubation, all the hatched chicks were assessed for chick quality of Tona score as described elsewhere (30), as chick quality was defined to encompass several qualitative characteristics and scored according to their importance, which could be a predictor of their later performance (30). Simply, the conditions of activity, down and appearance, retracted yolk, eyes, legs, navel area, remaining membrane, and remaining yolk were assessed and scored according to their importance within a total scale of 100 (30). The chicks were properly anesthetized with ethyl ether (Sinopharm Chemical Reagent Co., Ltd., Shanghai, China) after scoring before sampling. Then, the body weight (BW), yolk weight (YW), liver weight, and heart weight were measured by an electric balance (Shanghai Yingheng Electronic Scale Co., Ltd., Shanghai, China). The hatchability (%) was calculated as the ratio between the number of hatched chicks and the number of fertile eggs. The YFBM was calculated as the difference between BW and YW. The relative weight (%) of the heart and liver was calculated as the ratio between the liver or heart weight and the BW or YFBM.

This study was performed according to the Guidelines for the Care and Use of Laboratory Animals prepared by the Institutional Animal Care and Use Committee of Nanjing Agricultural University [Permit Number SYXK (Su) 2017-0007].

### Free amino acid analysis in the liver

A fully automatic amino acid analyzer (L-8080 type, Hitachi, Japan) was used to measure the free amino acid concentrations in the liver, according to the protocol described elsewhere (27). Six liver samples were randomly selected from each group for analysis. In brief, the liver samples were weighed and homogenized in a 5% sulfonic acid solution. After 30 min deproteinization on ice, the supernatant was filtered (0.22-μm filter, Biosharp, Guangzhou Saiguo Biotech Co., Ltd., Guangzhou, China) after 20 min of centrifugation (4°C, 20,000 *g*). The filtrate and standard solution were incorporated into the amino acid analyzer. The hepatic amino acid concentrations were expressed as pmol/mg on a wet tissue basis.

### Statistical analysis

Since there were two factors (embryonic days and TM) in Experiment 1, data were statistically analyzed by two-way analysis of variance (ANOVA). Holm–Sidak's multiple comparisons test was applied as a *post-hoc* analysis when a significant interaction was detected. The data in Experiment 2 were statistically analyzed by one-way ANOVA and Turkey's multiple comparisons test was applied when a significant difference was detected. Statistical analyses were performed using a commercially available package—GraphPad Prism 8 (GraphPad Software Inc., San Diego, CA). Significant differences were denoted as *P* < 0.05. Data were expressed as mean ± SEM. All data in each group were first subjected to outlier identification (*P* < 0.01) by GraphPad Prism 8, and the remaining data were used for the analysis among groups. The number of chicks used for statistical analysis in each group is shown in the Figure legends and Table notes.

## Results

### Influence of TM on embryonic growth during incubation

**Figure 2:**
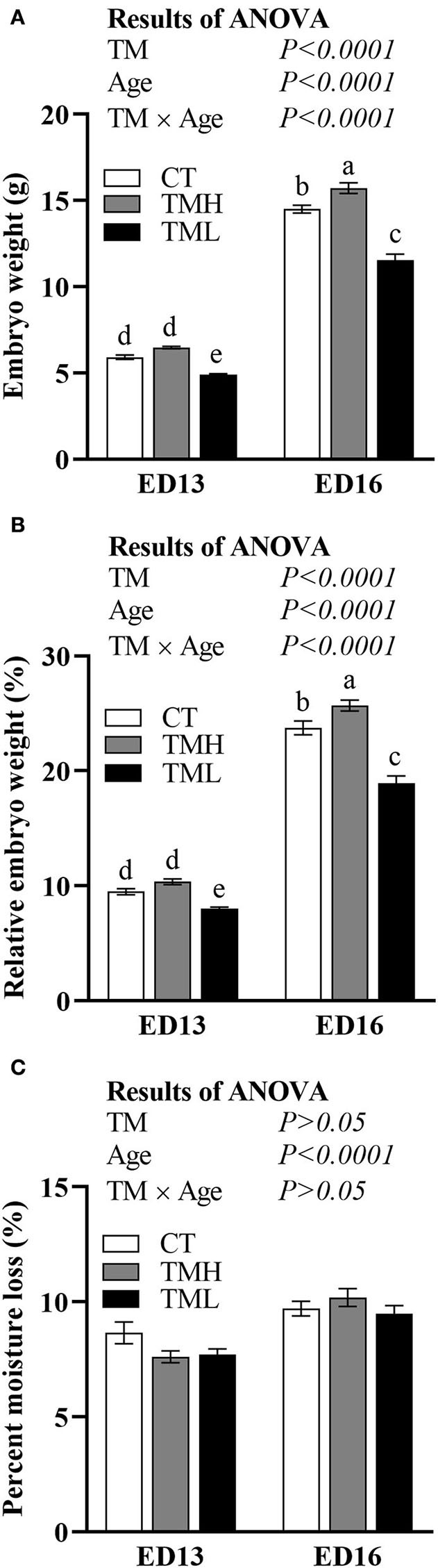
The changes in embryonic weight **(A)**, relative embryonic weight **(B)**, and percent moisture loss **(C)** exposed to control temperature (CT, 37.6°C) and TM with high temperature (TMH, 39°C, 8 h/day) or low temperature (TML, 20°C, 1 h/day) from embryonic day (ED) 8 to ED 15. The number of chicks in each group was *n* = 12–15. Values are mean ± SEM. Different superscripts indicate significant (*P* < 0.05) differences. TM, thermal manipulation.

The changes in embryonic weight, relative embryonic weight, and moisture loss are shown in Figure 2. The embryonic weight and relative embryonic weight were significantly (*P* < 0.0001) increased by the day of embryonic age and significantly (*P* < 0.0001) influenced by TM. A significant (*P* < 0.0001) interaction between embryonic age and TM suggested that the declined embryonic weight and relative embryonic weight by TML at ED 13 were fortified at ED 16. Moreover, the embryonic weight and relative embryonic weight were significantly improved by TMH at ED 16 but not at ED 13 (Figures 2A,B). The moisture loss in incubation was significantly increased with embryonic growth but was not affected by TM treatments (Figure 2C).

### Influence of TM on free amino acid concentrations in the liver of embryos

The changes in free amino acid concentrations in the liver of layer-type embryos are shown in Table 1. The hepatic free amino acid pool was enlarged with the progress of embryonic age since the concentration for all the amino acids was significantly (*P* < 0.01) increased. Except for histidine and glycine, the concentration of other essential amino acids (leucine, valine, isoleucine, arginine, and so on) in the chicken as well as non-essential amino acids was significantly (*P* < 0.05) influenced by TM treatment. Significant interactions between age and TM suggested that most of the influenced amino acids were significantly (*P* < 0.05) declined by TM treatment at ED 13 but not at ED 16. Interestingly, the treatments of TMH and TML had a similar type of effects with declining hepatic amino acid concentrations at ED 13.

**Table 1:** Free amino acid contents in the liver of embryos exposed to control or modified temperature during embryogenesis.

**Amino acids**	**ED13**			**ED16**			***P*** **value**		
	**CT**	**TMH**	**TML**	**CT**	**TMH**	**TML**	**Age**	**TM**	**Interaction**
**Essential amino acids**
Histidine	524 ± 138	167 ± 24	100 ± 21	822 ± 252	674 ± 218	735 ± 226	^**^	NS	NS
Threonine	5,788 ± 1,507^b^	1,663 ± 271^c^	1,066 ± 118^c^	9,656 ± 452^a^	10,151 ± 680^a^	9,604 ± 438^a^	^****^	^**^	^**^
Arginine	847 ± 172^a^	178 ± 36^b^	145 ± 18^b^	956 ± 76^a^	899 ± 86^a^	1,270 ± 162^a^	^****^	^**^	^***^
Valine	918 ± 238^a^	297 ± 40^b^	261 ± 30^b^	1,257 ± 75^a^	1,319 ± 6^a^	1,123 ± 44^a^	^****^	^**^	^*^
Isoleucine	425 ± 88^b^	125 ± 17^c^	118 ± 5^c^	616 ± 31^a^	605 ± 25^a^	588 ± 37^ab^	^****^	^***^	^**^
Methionine	260 ± 59^b^	79 ± 11^c^	64 ± 10^c^	469 ± 35^a^	477 ± 14^a^	452 ± 23^a^	^****^	^**^	^**^
Leucine	760 ± 173^b^	223 ± 29^c^	175 ± 22^c^	1,484 ± 86^a^	1,499 ± 43^a^	1,431 ± 57^a^	^****^	^**^	^**^
Lysine	1,246 ± 180^b^	293 ± 44^c^	268 ± 36^c^	2,879 ± 166^a^	3,056 ± 201^a^	3,046 ± 139^a^	^****^	^*^	^***^
Glycine	1,654 ± 366^b^	558 ± 90^c^	549 ± 64^c^	2,743 ± 154^a^	2,993 ± 142^a^	3,129 ± 144^a^	^****^	NS	^**^
Phenylalanine	494 ± 94^b^	136 ± 17^c^	99 ± 15^c^	922 ± 52^a^	892 ± 44^a^	811 ± 33^a^	^****^	^****^	^**^
**Non-essential amino acids**
Aspartic acid	1,284 ± 322^a^	456 ± 30^b^	245 ± 35^b^	1,700 ± 92^a^	1,692 ± 13^a^	1,934 ± 184^a^	^****^	^*^	^**^
Tyrosine	807 ± 218	222 ± 30	235 ± 30	887 ± 159	952 ± 173	731 ± 147	^***^	^*^	NS
Cystathionine	104 ± 31^b^	23 ± 4^c^	20 ± 5^c^	169 ± 11^a^	180 ± 15^a^	126 ± 10^ab^	^****^	^**^	^*^
Glutamic acid	7,126 ± 1,667^a^	2,005 ± 264^b^	2,238 ± 258^b^	7,318 ± 179^a^	7,343 ± 547^a^	6,920 ± 441^a^	^****^	^**^	^**^
Serine	2,764 ± 638^a^	777 ± 137^b^	563 ± 57^b^	3,303 ± 118^a^	3,890 ± 218^a^	3,728 ± 253^a^	^****^	^*^	^***^
Ammonia	4,133 ± 1,085^b^	1,286 ± 181^c^	1,246 ± 92^c^	7,684 ± 196^a^	7,755 ± 218^a^	8,262 ± 682^a^	^****^	^*^	^*^
Alanine	1,559 ± 399^b^	426 ± 62^c^	302 ± 43^c^	2,397 ± 142^a^	2,497 ± 74^a^	2,385 ± 112^a^	^****^	^**^	^**^

The number of embryos used in each group was as n = 5–6. Different superscripts in the same row indicate significant differences (P < 0.05) between treatments. Values are means ± SEM in pmol/mg. CT, control treatment (37.6°C) during incubation; TMH, thermal manipulation with higher incubation temperature (39°C during ED 8–15 with daily 8-h treatment from 10:00 to 18:00) during incubation; TML, thermal manipulation with lower incubation temperature (20°C during ED 8–15 with daily 1-h treatment from 14:00 to 15:00) during incubation; ED, embryonic day.

* P < 0.05.

** P < 0.01.

*** P < 0.001.

**** P < 0.0001.

NS, not significant.

### Influence of TM on hatching performance in layer-type chicks

**Figure 3:**
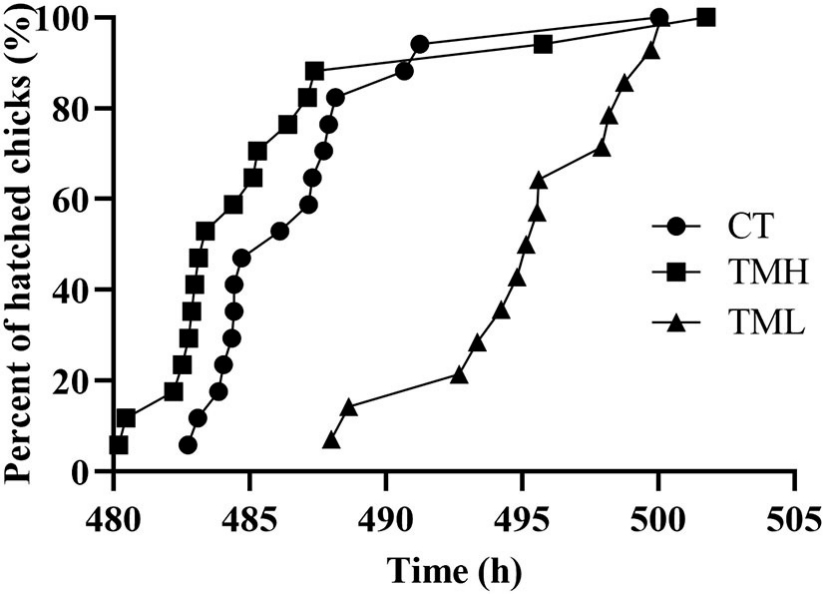
Distribution of hatch after exposure to control temperature (CT, 37.6°C) and thermal manipulation with high temperature (TMH, 39°C, 8 h/day) or low temperature (TML, 20°C, 1 h/day) from embryonic day (ED) 8 to ED 15. The Y axis is percent of hatched birds and the X axis is the incubation duration.

The results of the hatching process, incubation duration, and hatching performance are shown in Figure 3 and Tables 2, 3, respectively. Each group has 19 fertile eggs for hatching, and the number of hatched chicks was 16, 17, and 14 for CT, TMH, and TML, respectively, and one bird in the TMH group was dead after hatching. The hatchability was 84.2, 89.5, and 73.7% for CT, TMH, and TML, respectively. The hatchability in the TML group was clearly lower than in the other two groups. The chick of the TMH group first hatched was 2.55 h earlier than that of the CT group (482.73 h for CT; 480.18 h for TMH); however, the THL treatment delayed 5.27 h for first hatching (488.00 h for TML) compared with the CT group (Figure 3). The incubation duration was significantly (*P* < 0.05) longer in the THL group than in CT and TMH groups, and the THL treatment delayed incubation time 35–46 min/day for hatching after ED 8 in comparison with CT or TMH groups (Table 2). At hatching, TM treatments did not show significant effects on the BW, YW, YFBM, and chick quality (Tona score; Table 3). The weight of the heart and liver in chicks was also not affected by TMH or TML, even TML showed a trend of decline in liver weight (*P* = 0.0596 for CT vs. TML) at hatching. Importantly, the ratio of liver weight to BW or YFBM was significantly (*P* < 0.05) declined by the TML treatment at hatching.

**Table 2:** Incubation duration and calculated time delay (min/d) of hatching after exposure to control or modified temperature during embryogenesis.

**Spread of hatch (%)**	**Incubation duration (h)**			**Delay from ED 8**	
	**CT**	**TMH**	**TML**	**THL-CT**	**THL-TMH**
50	484.2 ± 0.3^a^	482.3 ± 0.4^a^	492.4 ± 1.1^b^	35	43
75	485.2 ± 0.5^a^	483.2 ± 0.5^a^	494.0 ± 1.0^b^	38	46
100	486.9 ± 1.0^a^	485.5 ± 1.3^a^	495.2 ± 1.0^b^	36	42

Different superscripts in the same row indicate significant differences (P < 0.05) between treatments. Values are means ± SEM. CT, control treatment (37.6°C) during incubation; TMH, thermal manipulation with higher incubation temperature (39°C during ED 8–15 with daily 8-h treatment from 10:00 to 18:00) during incubation; TML, thermal manipulation with lower incubation temperature (20°C during ED 8–15 with daily 1-h treatment from 14:00 to 15:00) during incubation; ED, embryonic day.

**Table 3:** Hatching performance after exposure to control or modified temperature during embryogenesis.

**Items**	**CT**	**TMH**	**TML**	***P-*value**
BW (g)	40.64 ± 0.93	39.05 ± 1.10	42.99 ± 1.28	NS
YW (g)	4.53 ± 0.29	4.12 ± 0.27	5.15 ± 0.36	NS
YFBM (g)	36.11 ± 0.79	34.93 ± 0.87	37.65 ± 1.00	NS
Liver (g)	0.98 ± 0.03	0.95 ± 0.02	0.89 ± 0.03	NS
Heart (g)	0.29 ± 0.01	0.30 ± 0.01	0.31 ± 0.01	NS
Tona-score	90.6 ± 1.1	92.0 ± 1.5	91.4 ± 0.8	NS
Liver/BW (%)	2.39 ± 0.09^a^	2.45 ± 0.05^a^	2.06 ± 0.07^b^	^**^
Heart/BW (%)	0.73 ± 0.03	0.77 ± 0.02	0.65 ± 0.05	NS
Liver/YFBM (%)	2.69 ± 0.11^a^	2.73 ± 0.05^a^	2.35 ± 0.07^b^	^**^
Heart/YFBM (%)	0.82 ± 0.03	0.85 ± 0.26	0.75 ± 0.06	NS

The number of embryos used in each group was n = 8–10. Different superscripts in the same row indicate significant differences (P < 0.05) between treatments. Values are means ± SEM. CT, control treatment (37.6°C) during incubation; TMH, thermal manipulation with higher incubation temperature (39°C during ED 8–15 with daily 8-h treatment from 10:00 to 18:00) during incubation; TML, thermal manipulation with lower incubation temperature (20°C during ED 8–15 with daily 1-h treatment from 14:00 to 15:00) during incubation; ED, embryonic day; BW, body weight; YW, yolk weight; YFBM, yolk free body mass.

** P < 0.01.

NS, not significant.

## Discussion

The objective of this study was to investigate embryonic growth, changes in hepatic amino acid metabolism, and hatching performance after TM with high or low incubation temperature in layer-type chickens. Temperature manipulation during the middle stages caused a significant modification in embryonic growth, incubation duration, hepatic amino acid concentrations, and several parameters of hatched chicks in comparison with the control group.

Temperature manipulation is considered a management tool to influence embryonic development. In the current study, the decreased incubation temperature induced significant retardation in embryo growth at ED 13 and 16. The threshold temperature for embryonic development is 27°C and embryonic development stops below at ~27°C (31). Shinder et al. (10) reported that the heart rate of embryos was reduced from 252 to 35 beats/min after 60 min cold exposure (15°C) and recovered under control incubation temperature. The cold exposure to 20°C for 60 min was thought to delay embryonic development for a short term, and 5 days (or less) accumulation of TML treatment resulted in lower embryonic weight. However, the hatchability, which is a critical parameter for hatchery managers, was clearly lower in the TML treatment than in the control group in this study. Our recent study agrees with these results that TML treatment of 20°C for 60 min reduced hatchability by 11% in layer chicks (unpublished data). It indicated that 20°C for 60 min might be too harsh for layer embryos, even 15°C for 60 min caused no negative effects on hatchability in broiler chicks (10). On the contrary, TMH treatment (39°C for 8 h/d during ED 8–15) was expected to accelerate embryonic development and increased the embryo's relative weight at ED 16, which agreed with the previous report that TM with 39.5°C for 12 h/d during ED 7–16 significantly increased embryonic relative weight at ED 11, 12, 14, and 16 in broiler strain (32). The threshold of heat injury is 40.5°C, and no embryos hatched under continuous temperatures above 40.5°C (31). However, the short periods of high temperature are not necessarily lethal for embryos. A continuous 40.6°C between ED 16 and 18.5 resulted in lower embryo weight and higher embryonic mortality (24). The hypothalamic-pituitary-adrenal and hypothalamic-pituitary-thyroid axes are the main efferent neuro-hormonal axis for stress responses and metabolic regulation (1, 33), which are developed from ED7 to ED16 in chick embryos (7, 28). Elevated incubation temperature during the middle stages (ED 7–ED 16) promoted myoblast proliferation and muscle growth in broiler embryos, as well as caused a long-term effect on post-hatch broiler chicken (34). As mentioned above, TM treatments during ED 8–15 significantly affected the embryo growth in layer-type chicks. However, embryo growth and physiological development are different between layer and broiler strains (14, 35), as different purposes of intensive selection. Then, the most appreciated period or “critical” developmental period by TM treatments, as well as the suitable temperature for cold stimulation needs to be further investigated in layer strains.

The effects of TM, cold, or heat, in embryonic growth, are controversial due to the differences in temperature, duration, and embryonic stages of TM treatments. However, it is unanimous that TM of low or high temperature could affect the physiology and anti-stress ability of post-hatch chicks or chickens in a long term (12, 36, 37). Thermal manipulation with high temperature (TMH) has been demonstrated to afford thermotolerance to later heat exposure in broiler chickens (1, 38). Cyclic cold exposure during incubation caused to increase cold resistance in broilers (36). Moreover, embryonic TM was also reported to afford cross-adaptation to stress in broilers (39). In the present study, TMH and TML induced opposite effects on embryonic growth, thus, it is possible to apply both TMH and TML to regulate embryonic growth and to improve the acquisition of cold, heat, or cross resistance with avoiding harmful consequences of TM treatments in post-hatch chickens. Therefore, the collective effects of TMH and TML during incubation on physiology, growth, and stress responses in layer-type chicks and hens are needed to be investigated in future studies.

The hatching process and day-old chick quality are affected by the incubation environment and egg characteristics (30, 40). Although both TMH and TML significantly affected embryonic growth, only TML significantly delayed the incubation duration. However, there was no significant effect of TM treatments on the speed of hatching and Tona score. The YFBM and BW of day-old chick are important parameters of day-old chick quality and closely related to slaughter weight in broilers (41), but the relationship between YFBM (or BW) and laying performance is still unclear in layer hens. The higher eggshell temperature during incubation significantly decreased YFBM with 0.7 g at hatching in layer-type chicks, which was contributed by improved percent moisture loss under high incubation temperature (42). Although significant differences were not detected, the average YFBM in the TMH group declined to 1.18 g compared with the control group. Interestingly, both TMH and TML caused significant effects on embryo growth on ED 16, but no differences were found at hatching. The body mass is correlated with egg weight, and percent moisture loss was not affected by TM treatments in the current study. Similar results were reported that *in ovo* feeding of L-Leu caused growth retardation of embryos but no effects on body weight at hatching in broilers (21, 27). However, the laying performance and eggshell quality are more concerned than slaughter weight in layer-type strains. Thus, TM-mediated effects on the laying performance of hens should be clarified in the future.

During incubation, the free amino acids play an important role in layer- and meat-type embryonic growth (14). The free amino acid concentrations were increased during incubation, which agrees with previous reports that the albumin uptake into the embryo was increased and the free amino acid pool was enlarged to match the amino acid requirements of embryogenesis (21, 43). Our previous study demonstrated that TMH decreased some essential amino acid concentrations in the embryos and the altered amino acid was demonstrated to afford thermotolerance in broiler chicks and chickens (20–22). Similarly, most amino acids declined under TMH in the embryonic liver. The declined hepatic amino acids are speculated to use to cope with the increased incubation temperature, which has been demonstrated in broiler strains, as L-Leu *in ovo* feeding affords thermotolerance (22). Interestingly, the TML showed similar effects with declining hepatic amino acid concentrations. One possibility could be that the decreased free amino acids played the same roles in coping with cold exposure in developing embryos, or the nutritional mechanisms of heat- or cold-resistance were similar in poultry. Both heat and cold exposures are identified as thermal stress. During stressful conditions, some amino acids are used to attenuate stress response in the chicken (44). Promoted protein synthesis or gluconeogenic phenomenon under high incubation temperature might contribute to the decline of free amino acid in the liver (45). Saleh et al. (38) reported that TMH caused to increase in body weight and declined body temperature after both cold and heat stress in broiler chickens. Epigenetic modification or improved oxidative function might be the reasons for TM-mediated cross adaptation to later stress in post-hatch broilers (38, 39). In addition, TML caused retardation in embryonic growth, which might be another contributor to declined amino acid concentrations in the TML group, as the free amino acid pool is correlated with stages of embryonic development. However, the TM-mediated difference in the concentration of free amino acid disappeared after TM treatment at ED 16, even though the growth was significantly slowed down in the TML group and improved in the TMH group. This is consistent with our previous report that the TMH treatment (38.6°C for 6 h/d during ED 10–18) caused a decline in some hepatic amino acid levels at ED 14 but not at ED 19, in broiler embryos (21). The liver plays an important role in amino acid metabolism; however, the brain was considered the center of thermoregulation (33). The effects of TM treatments on amino acid metabolism in the blood, brain, and other tissues of layer embryos need to be investigated in the future, which is expected to provide important clues for the potential agent (s) in affording stress-resistance in layer chicks and hens.

## Conclusion

In summary, TM of high or low incubation temperature during the middle stages of embryogenesis caused different modifications in embryonic growth, incubation duration, and hatching process in layer strain. Interestingly, the changes in hepatic amino acid concentrations were similar after both cold and heat exposure in layer embryos. Future studies will investigate the effects of TM treatments on post-hatch performance in layer-type chicks and hens.

## Data availability statement

The original contributions presented in the study are included in the article/supplementary material, further inquiries can be directed to the corresponding author.

## Ethics statement

The animal study was reviewed and approved by the Institutional Animal Care and Use Committee of Nanjing Agricultural University.

## Author contributions

GH and CL designed this research. GH, SL, and YL conducted animal experiments. GH, SL, PT, and TB performed the sample analysis and statistical analysis. GH and VC wrote the manuscript. MF, ZB, and CL reviewed and edited the manuscript. All authors read and approved the final manuscript.

## Funding

This study was partly supported by grants from the Jiangsu Agricultural Industry Technology System (Grant No. JATS [2022] 480), the Jiangsu Agriculture Science and Technology Innovation Fund [Grant No. CX(22)1008], Jiangsu Modern Agricultural Machinery Equipment and Technology Demonstration and Promotion Project (Grant No. NJ2021-25), and North Jiangsu Science and Technology Project (Grant No. XZ-SZ202119), JAAS Fund for International Cooperation.

## Conflict of interest

The authors declare that the research was conducted in the absence of any commercial or financial relationships that could be construed as a potential conflict of interest.

## Publisher's note

All claims expressed in this article are solely those of the authors and do not necessarily represent those of their affiliated organizations, or those of the publisher, the editors and the reviewers. Any product that may be evaluated in this article, or claim that may be made by its manufacturer, is not guaranteed or endorsed by the publisher.

## References

[1] Loyau T Bedrani L Berri C Metayer-Coustard S Praud C Coustham V. Cyclic variations in incubation conditions induce adaptive responses to later heat exposure in chickens: a review. Animal. (2015) 9:76–85. 10.1017/S1751731114001931 25118598

[2] Whittow GC Tazawa H. The early development of thermoregulation in birds. Physiol Biochem Zool. (1991) 64:1371–90. 10.1086/physzool.64.6.30158220

[3] Boleli IC Morita VS Matos JB Jr., Thimotheo M, Almeida VR. Poultry egg incubation: integrating and optimizing production efficiency. Braz J Poult Sci. (2016) 18:1–16. 10.1590/1806-9061-2016-0292

[4] Hulet RM. Symposium: managing the embryo for performance managing incubation: where are we and why? Poult Sci. (2007) 86:1017–9. 10.1093/ps/86.5.1017 17435041

[5] Moraes VMB Malheiros RD Bruggeman V Collin A Tona K Van As P. Effect of thermal conditioning during embryonic development on aspects of physiological responses of broilers to heat stress. J Therm Biol. (2003) 28:133–40. 10.1016/S0306-4565(02)00049-9

[6] Loyau T Metayer-Coustard S Berri C Crochet S Cailleau-Audouin E Sannier M. Thermal manipulation during embryogenesis has long-term effects on muscle and liver metabolism in fast-growing chickens. PLoS ONE. (2014) 9:e105339. 10.1371/journal.pone.0105339 25180913 PMC4152147

[7] Piestun Y Shinder D Ruzal M Halevy O Yahav S. The effect of thermal manipulations during the development of the thyroid and adrenal axes on in-hatch and post-hatch thermoregulation. J Therm Biol. (2008) 33:413–8. 10.1016/j.jtherbio.2008.06.007

[8] He SP Arowolo MA Medrano RF Li S Yu QF Chen JY. Impact of heat stress and nutritional interventions on poultry production. Worlds Poult Sci J. (2018) 74:647–64. 10.1017/S004393391800072733025116

[9] Kamanli S Yalçin ZS TaşdemIr AN Tarim B Tülek E Aygören H. Effect of eggshell temperatures on hatching performance, egg production, and bonemorphology of laying hens. Turk J Vet Anim Sci. (2021) 45:11–20. 10.3906/vet-2006-31

[10] Shinder D Ruzal M Giloh M Druyan S Piestun Y Yahav S. Improvement of cold resistance and performance of broilers by acute cold exposure during late embryogenesis. Poult Sci. (2011) 90:633–41. 10.3382/ps.2010-01089 21325235

[11] Akşit M Yalçin S Siegel PB Yenisey Ç Özdemir D Özkan SEZEN. Broilers respond to cooler ambient temperatures after temperature acclimation during incubation and early postnatal age. J Appl Poult Res. (2013) 22:298–307. 10.3382/japr.2012-00675

[12] Nyuiadzi D Berri C Dusart L Travel A Méda B Bouvarel I. Short cold exposures during incubation and postnatal cold temperature affect performance, breast meat quality, and welfare parameters in broiler chickens. Poult Sci. (2020) 99:857–68. 10.1016/j.psj.2019.10.024 32029166 PMC7587810

[13] Kamanli S Durmuş I Yalçin S Yildirim U Meral Ö. Effect of prenatal temperature conditioning of laying hen embryos: Hatching, live performance and response to heat and cold stress during laying period. J Therm Biol. (2015) 51:96–104. 10.1016/j.jtherbio.2015.04.001 25965022

[14] Sato M Tomonaga S Denbow DM Furuse M. Changes in free amino acids in the brain during embryonic development in layer and broiler chickens. Amino Acids. (2009) 36:303–8. 10.1007/s00726-008-0068-z 18389170

[15] Ohta Y Kidd MT Ishibashi T. Embryo growth and amino acid concentration profiles of broiler breeder eggs, embryos, and chicks after *in ovo* administration of amino acids. Poult Sci. (2001) 80:1430–6. 10.1093/ps/80.10.1430 11599701

[16] Baker DH. Advances in protein–amino acid nutrition of poultry. Amino Acids. (2009) 37:29–41. 10.1007/s00726-008-0198-3 19009229

[17] Chowdhury VS Tomonaga S Ikegami T Erwan E Ito K Cockrem JF. Oxidative damage and brain concentrations of free amino acid in chicks exposed to high ambient temperature. Comp Biochem Physiol Part A Mol Integr Physiol. (2014) 169:70–6. 10.1016/j.cbpa.2013.12.020 24389089

[18] Ito K Erwan E Nagasawa M Furuse M Chowdhury VS. Changes in free amino acid concentrations in the blood, brain and muscle of heat-exposed chicks. Br Poult Sci. (2014) 55:644–52. 10.1080/00071668.2014.957653 25157850

[19] Han G Yang H Wang Y Zhang R Tashiro K Bungo T. Effects of *in ovo* feeding of L-leucine on amino acids metabolism and heat-shock protein-70, and−90 mRNA expression in heat-exposed chicks. Poult Sci. (2019) 98:1243–53. 10.3382/ps/pey444 30265371

[20] Han G Ouchi Y Hirota T Haraguchi S Miyazaki T Arakawa T. Effects of l-leucine *in ovo* feeding on thermotolerance, growth and amino acid metabolism under heat stress in broilers. Animal. (2020) 14:1701–9. 10.1017/S175173112000046433490137

[21] Han G Yang H Bahry MA Tran PV Do PH Ikeda H. l-Leucine acts as a potential agent in reducing body temperature at hatching and affords thermotolerance in broiler chicks. Comp Biochem Physiol Part A Mol Integr Physiol. (2017) 204:48–56. 10.1016/j.cbpa.2016.10.013 27840178

[22] Han G Yang H Bungo T Ikeda H Wang Y Nguyen LT. *In ovo* L -leucine administration stimulates lipid metabolisms in heat-exposed male, but not female, chicks to afford thermotolerance. J Therm Biol. (2018) 71:74–82. 10.1016/j.jtherbio.2017.10.020 29301703

[23] Ipek AYDIN Sahan U Baycan SC Sozcu ARDA. The effects of different eggshell temperatures on embryonic development, hatchability, chick quality, and first-week broiler performance. Poult Sci. (2014) 93:464–72. 10.3382/ps.2013-03336 24570470

[24] Willemsen H Kamers B Dahlke F Han H Song Z Pirsaraei ZA. High- and low-temperature manipulation during late incubation: effects on embryonic development, the hatching process, and metabolism in broilers. Poult Sci. (2010) 89:2678–90. 10.3382/ps.2010-00853 21076107

[25] Sgavioli S Matos Júnior JB Borges LL Praes MFFM Morita VS Zanirato GL. Effects of ascorbic acid injection in incubated eggs submitted to heat stress on incubation parameters and chick quality. Braz J Poult Sci. (2015) 17:181–9. 10.1590/1516-635x1702181-190

[26] Willemsen H Everaert N Witters A De Smit L Debonne M Verschuere F. Critical assessment of chick quality measurements as an indicator of posthatch performance. Poult Sci. (2008) 87:2358–66. 10.3382/ps.2008-00095 18931188

[27] Han G Ren Y Shen D Li S Chowdhury VS Li Y. L-Leucine *in ovo* administration causes growth retardation and modifies specific amino acid metabolism in broiler embryos. J Poult Sci. (2021) 58:163–70. 10.2141/jpsa.0200086 34447280 PMC8371536

[28] Reyns GE Venken K de Escobar GM Kühn ER Darras VM. Dynamics and regulation of intracellular thyroid hormone concentrations in embryonic chicken liver, kidney, brain, and blood. Gen Comp Endocr. (2003) 134:80–7. 10.1016/S0016-6480(03)00220-X 13129506

[29] Al-Zghoul MB Saleh KMM Jaradat ZW. Expression of digestive enzyme and intestinal transporter genes during chronic heat stress in the thermally manipulated broiler chicken. Poult Sci. (2019) 98:4113–22. 10.3382/ps/pez249 31065718

[30] Tona K Bamelis F De Ketelaere B Bruggeman V Moraes VM Buyse J. Effects of egg storage time on spread of hatch, chick quality, and chick juvenile growth. Poult Sci. (2003) 82:736–41. 10.1093/ps/82.5.736 12762394

[31] Decuypere E Michels H. Incubation temperature as a management tool: a review. Worlds Poult Sci J. (1992) 48:28–38. 10.1079/WPS19920004

[32] Piestun Y Halevy O Yahav S. Thermal manipulations of broiler embryos–the effect on thermoregulation and development during embryogenesis. Poult Sci. (2009) 88:2677–88. 10.3382/ps.2009-00231 19903968

[33] Yahav S. Regulation of body temperature: strategies and mechanisms. In:Scanes C, editors. Sturkie's Avian Physiology, 6th ed. London: Acad. Press. (2015). p. 869–905.

[34] Piestun Y Yahav S Halevy O. Thermal manipulation during embryogenesis affects myoblast proliferation and skeletal muscle growth in meat-type chickens. Poult Sci. (2015) 94:2528–36. 10.3382/ps/pev245 26316337

[35] Sato M Tachibana T Furuse M. Heat production and lipid metabolism in broiler and layer chickens during embryonic development. Comp Biochem Physiol Part A Mol Integr Physiol. (2006) 143:382–8. 10.1016/j.cbpa.2005.12.019 16460976

[36] Yalcin S Özkan S Siegel P Yenisey Ç Aksit M. Manipulation of incubation temperatures to increase cold resistance of broilers:influence on embryo development, organ weights, hormones and body composition. J Poult Sci. (2012) 49:133–9. 10.2141/jpsa.011117

[37] Loyau T Berri C Bedrani L Metayer-Coustard S Praud C Duclos MJ. Thermal manipulation of the embryo modifies the physiology and body composition of broiler chickens reared in floor pens without affecting breast meat processing quality. J Anim Sci. (2013) 91:3674–85. 10.2527/jas.2013-6445 23736053

[38] Saleh KM Tarkhan AH Al-Zghoul MB. Embryonic thermal manipulation affects the antioxidant response to post-hatch thermal exposure in broiler chickens. Animals. (2020) 10:126. 10.3390/ani10010126 31941014 PMC7022970

[39] Rosenberg T Kisliouk T Cramer T Shinder D Druyan S Meiri N. Embryonic heat conditioning induces tet-dependent cross-tolerance to hypothalamic inflammation later in life. Front Genet. (2020) 11:767. 10.3389/fgene.2020.00767 32849788 PMC7419591

[40] Deeming DC. Incubation and chick rearing. In:Glatz P Lunam C Malecki I, editors. The Welfare of Farmed Ratites. Berlin, Heidelberg: Springer (2011). p. 65–89.

[41] da Silva CS Molenaar R Giersberg MF Rodenburg TB van Riel JW De Baere K. Day-old chicken quality and performance of broiler chickens from 3 different hatching systems. Poult Sci. (2021) 100:100953. 10.1016/j.psj.2020.12.050 33518300 PMC7936180

[42] Molenaar R de Vries S van den Anker I Meijerhof R Kemp B van den Brand H. Effect of eggshell temperature and a hole in the air cell on the perinatal development and physiology of layer hatchlings. Poult Sci. (2010) 89:1716–23. 10.3382/ps.2010-00779 20634528

[43] Ohta Y Yoshida T Tsushima N. Comparison between broilers and layers for growth and protein use by embryos. Poult Sci. (2004) 83:783–7. 10.1093/ps/83.5.783 15141836

[44] Furuse M. Screening of central functions of amino acids and their metabolites for sedative and hypnotic effects using chick models. Eur J Pharmacol. (2015) 762:382–93. 10.1016/j.ejphar.2015.06.036 26101060

[45] Kelley PM Schlesinger MJ. The effect of amino acid analogues and heat shock on gene expression in chicken embryo fibroblasts. Cell. (1978) 15:1277–86. 10.1016/0092-8674(78)90053-3 569556

